# Body fat distribution and bone mineral density in a multi-ethnic sample of postmenopausal women in The Malaysian Cohort

**DOI:** 10.1007/s11657-024-01435-x

**Published:** 2024-08-07

**Authors:** Holly Bihun, Noraidatulakma Abdullah, Nor Azian Abdul Murad, Siok Fong Chin, Azwa Shawani Kamalul Arifin, Aisyatul Najihah Khuzaimi, Fredrik Karpe, Sarah Lewington, Jennifer Carter, Fiona Bragg, Rahman Jamal

**Affiliations:** 1 Clinical Trial Service Unit and Epidemiological Studies, Nuffield Department of Population Health, Big Data Institute, University of Oxford, Richard Doll Building, Old Road Campus, Oxford, OX3 7LF UK; 2 UKM Medical Molecular Biology Institute (UMBI), UKM KL Campus, Jalan Yaacob Latiff, Cheras, 56000 Kuala Lumpur, Malaysia; 3 Oxford Centre for Diabetes, Endocrinology and Metabolism, University of Oxford, Churchill Hospital, Headington, OX3 7LE UK; 4 Health Data Research UK, University of Oxford (HDRUK-Oxford), Oxford, UK

**Keywords:** Adiposity, Bone mineral density, Dual X-ray absorptiometry, Postmenopausal women, Ethnicity, Cross-sectional

## Abstract

**Summary:**

In this study of postmenopausal women in Malaysia, total adiposity was inversely associated with total BMD, while regional associations varied. No differences were detected across Malay, Chinese, and Indian ethnicities. Low BMD contributes substantially to morbidity and mortality, and increasing adiposity levels globally may be contributing to this.

**Purpose:**

To investigate associations of total and regional adiposity with bone mineral density (BMD) among a multi-ethnic cohort of postmenopausal women.

**Methods:**

Dual X-ray absorptiometry (DXA) imaging was undertaken for 1990 postmenopausal women without prior chronic diseases (30% Malay, 53% Chinese, and 17% Indian) from The Malaysian Cohort (TMC). The strength of the associations between standardized total and regional body fat percentages with total and regional BMD was examined using linear regression models adjusted for age, height, lean mass, ethnicity, education, and diabetes. Effect modification was assessed for ethnicity.

**Results:**

Women with a higher total body fat percentage were more likely to be Indian or Malay. Mean (SD) BMD for the whole-body total, lumbar spine, leg, and arm were 1.08 (0.11), 0.96 (0.15), 2.21 (0.22), and 1.36 (0.12) g/cm^2^, respectively. Total body and visceral fat percentage were inversely associated with total BMD (− 0.02 [95% CI − 0.03, − 0.01] and − 0.01 [− 0.02, − 0.006] g/cm^2^ per 1 SD, respectively). In contrast, subcutaneous and gynoid fat percentages were positively associated with BMD (0.007 [0.002, 0.01] and 0.01 [0.006, 0.02] g/cm^2^, respectively). Total body fat percentage showed a weak positive association with lumbar BMD (0.01 [0.004, 0.02]) and inverse associations with leg (− 0.04 [− 0.06, − 0.03]) and arm (− 0.02 [− 0.03, − 0.02]) BMD in the highest four quintiles. There was no effect modification by ethnicity (*p*_hetero_ > 0.05).

**Conclusion:**

Total adiposity was inversely associated with total BMD, although regional associations varied. There was no heterogeneity across ethnic groups suggesting adiposity may be a risk factor for low BMD across diverse populations.

**Supplementary Information:**

The online version contains supplementary material available at 10.1007/s11657-024-01435-x.

## Introduction

Low bone mineral density (BMD) defines osteoporosis as a metabolic disease responsible for significant disability globally [1], and its association with mortality is similar to that of blood pressure or serum cholesterol [2]. From 1990 to 2019, global disability-adjusted life years attributable to low BMD were estimated to have nearly doubled from 8.6 million (95% CI 7.04–10.14) to 16.6 million (13.50–20.04) with more deaths associated with low BMD fractures after the age of 40 among women than men [1]. The gender disparity of the health burden attributable to low BMD is greater at older ages [1] and is projected to continue to widen. Understanding modifiable risk factors for low BMD, including among postmenopausal women, is therefore important, and arguably increasingly so given the aging population.

Adiposity levels have been suggested to be one such modifiable risk factor for low BMD. A systematic review found that body mass index (BMI) was positively associated with BMD of the lumbar spine, femoral neck, radius, and hip in postmenopausal women [3]. However, the use of standard anthropometric measures such as BMI and waist and hip circumferences may be over-simplistic and less accurate as an estimate of adiposity [4]. For instance, these measures preclude understanding of the relevance of regional adiposity, and the distinct contributions of lean and fat mass on bone health cannot be distinguished since measures like BMI are a composite of those three body components. The use of more specific and accurate adiposity measures from dual X-ray absorptiometry (DXA) to investigate this association has produced conflicting results with regard to direction and strength [2, 5–17]. These differing findings may be due to investigation of the relationships of adiposity measures with BMD in small study populations [6–8, 10–12, 15–17] using different DXA body parameters (i.e., total and/or regional adiposity as an absolute tissue mass [2, 5–12, 15, 16], percentage of total or regional mass [2, 6, 10, 11, 13, 14, 17], or ratio [2, 7, 10, 11, 14]) that are derived from varied, and potentially inconsistent, DXA models and software. Furthermore, previous research has typically been conducted in ethnically homogenous populations, limiting understanding of the relevance of adiposity for observed ethnic differences in clinical outcomes attributable to low BMD such as those for fragility fractures which are greater than any other type of fracture [18].

Evidence based on DXA adiposity measures of a large, multi-ethnic cohort of postmenopausal women is needed to further explore their association with BMD and potential ethnic differences in an increasingly at-risk population. Malaysia offers a novel context for investigating these ethnic differences given the country’s large subpopulations of individuals of Malay, Chinese, and Indian ethnicities that have documented differences in adiposity [19]. Using data from The Malaysian Cohort (TMC), the primary aim of the current study is to examine the associations between total body fat and fat distribution—assessed through DXA—and total and site-specific BMD among postmenopausal women. The study’s secondary aims are to investigate potential ethnic differences in these associations.

## Methods

### Study population: The Malaysian Cohort (TMC)

Details of the TMC study design and methods have been described previously [20]. Briefly, TMC is a prospective cohort study of 119,555 adults aged 35 to 75 who were recruited between 2006 and 2020. Recruitment was from 151 locations (95 rural and 56 urban) across Malaysia through voluntary participation campaigns, targeted sampling, and cluster sampling (in rural areas) [20]. All participants provided written informed consent. Sociodemographic and health information as well as biophysical measurements and biospecimens were collected at baseline, and all participants were actively followed up to complete a questionnaire and undergo biophysical measurements similar to baseline every 5 years [21]. As of 2021, TMC has a follow-up rate of 42.7% [21]. Ethics approval was provided by the ethics committee of Universiti Kebangsaan Malaysia (Project Code: FF-205–2007).

### The dual-energy X-ray absorptiometry (DXA) substudy

In 2020, 11,475 surviving participants residing in Kuala Lumpur or the surrounding state of Selangor who had completed at least one follow-up after recruitment and who reported no active cancer diagnosis were randomly selected to be invited via telephone to participate in a DXA substudy (response rate, 54%). DXA scans were undertaken between September 2020 and May 2023. Participants with a medical implant (*n* = 575), height greater than 180 cm or weight greater than 110 kg (*n* = 40), ethnicity recorded as “other” (*n* = 10), or with other relevant contradictions to participation (i.e., claustrophobia) (*n* = 22) were not included in the DXA substudy. DXA scans were performed using two Hologic DXA models (Discovery A and Discovery W). Sociodemographic characteristics and medical histories were collected at the time of the DXA scan using a shortened version of the study’s baseline questionnaire. HbA1c (%) was measured using a blood sample collected at TMC follow-up and the high-performance liquid chromatography (HPLC) in the Variant™ II Turbo machine (Bio-Rad Laboratories Inc, USA).

### DXA measurements

Height and weight were measured manually and inputted into the DXA system prior to the scan. The DXA system software automatically calculated BMI (weight [kg]/height [m]^2^) from these measures. Absolute total, subcutaneous, visceral, and gynoid fat (kg) as well as total lean mass (kg), a measure of muscle mass, were derived from whole-body DXA scans. The Hologic system’s software automatically located the inner and outer margins of the abdominal wall on DXA images across the fourth lumbar vertebra. Using these margins, subcutaneous and visceral fat were then measured in a 5-cm band above the iliac crest (pelvic cut line). Visceral fat area was measured within the system-derived lateral limits of the inner margins of the abdominal wall, while subcutaneous fat area measurements were derived from the assessment of tissue from the outer margins of the abdominal wall to the edge of subcutaneous fat outside the abdominal muscle wall (Fig. S1). A multivariable model including the measured visceral fat area was then used to optimize the agreement of DXA measures with computed tomography imaging. Subcutaneous and visceral fat mass were then derived from this adjusted area based on the thickness of the original DXA slice (5 cm) and the known density of fat (0.9 g/cm^3^). Gynoid fat was measured using delineations marked by the system software based on the height of an individual’s android region, calculated as 20% of the distance from the pelvic horizontal line to the neckline. The gynoid region was defined as having an upper boundary 1.5 times the height of the android region below the pelvic horizontal line, extending to a lower boundary twice the height of the android region below the upper boundary, and within the lateral limits of the arm lines (Fig. S1).

Total body fat was converted to a percentage of whole-body mass, while regional fat (subcutaneous, visceral, and gynoid body fat) was converted to a percentage of total body fat mass to focus on the importance of the location and distribution of fat in the body (i.e., [subcutaneous body fat (kg) / total body fat mass (kg)] × 100).^1^ Both total and regional body fat percentages were categorized into quintiles. Then, to create comparable beta coefficients for continuous linear regression models, total and regional body fat percentages were standardized to a mean of 0 and a standard deviation of 1.

Regional BMDs were computed by the Hologic system software using the manufacturer-recommended BMD region delineations applied to measurements from the total body DXA scan. Whole-body total and lumbar spine BMD were automatically generated by the DXA systems, while manufacturer-generated sections were combined to create BMD of the leg (left and right leg) and arm (left and right arm). The DXA systems used defined the lumbar region of the spine (L1–L4) as the area between the system-generated “spine lines,” lines that are parallel to the vertebral column, but their lengths run between the T12–L1 joint and upper pelvic lines. Leg regions were defined as the areas below the left and right pelvic divider lines and confined within the leg lines running from the outer hip to the foot on each side of the body. Arm regions were defined as the areas below the neckline but outside the right and left chest and leg lines (Fig. S1).

### Statistical analyses

The prevalence and mean (SD) values of participant characteristics (for categorical and continuous variables, respectively) were calculated across fifths of total body, subcutaneous, visceral, and gynoid fat percentage*.* Univariable associations between total body fat percentage and total BMD were conducted using ANOVA. Additional covariates were tested for their potential as confounders through assessment of their univariable associations with total body fat percentage and total BMD using Pearson’s chi-square tests and ANOVA and included in the adjusted model if *p* < 0.05. Since age at, and years since, last menses were highly correlated (*r* = 0.77), years since last menses was not included in the fully adjusted model.

Height (cm), lean mass (kg), ethnicity (Malay, Chinese, and Indian), education (highest level completed—primary, secondary, or tertiary), and diabetes (yes or no) were sequentially added to age-adjusted models of each body fat percentage measure (total body, subcutaneous, visceral, and gynoid) and BMD to assess the individual effects of each confounder. Quintiles of total and regional body fat percentages were plotted against fully adjusted means of total and regional BMD (lumbar spine, leg, and arm) to assess the shape of the associations. If the association was approximately linear, a fully adjusted linear regression model estimated the strength of the continuous association between standardized total, subcutaneous, visceral, and gynoid body fat percentage with whole-body total, lumbar spine, leg, and arm BMD [2, 3, 5, 10, 11, 22].

To investigate potential effect modification, the linear associations between standardized total or regional body fat percentage and total BMD were assessed among subgroups of ethnicity (Malay, Indian, and Chinese), and heterogeneity was assessed through a likelihood ratio test comparing models with and without an interaction term. Effect modification in the association of total or regional body fat percentages and total BMD was assessed for the following: years since the last menses (< 3 years, 3–6 years, > 6 years), BMI category (underweight/normal, < 23 kg/m^2^; overweight, 23–27.5 kg/m^2^; obese, > 27.5 kg/m^2^), and diabetes status (yes or no). A false discovery rate (FDR) correction using the Benjamini-Hochburg method was used to account for multiple testing.

Statistical analyses were performed using STATA 17.0 (StataCorp LLC, College Station, TX, USA) and RStudio (Integrated Development for R. RStudio, PBC, Boston, MA).

## Results

### Participant characteristics

Of the 5489 DXA substudy participants, 3153 (57%) were women. After excluding 872 (28%) premenopausal women, 281 (9%) with relevant medical conditions (chronic hepatitis, chronic bronchitis, cancer within the past 5 years, pulmonary tuberculosis, kidney disease, or asthma) and 10 (0.3%) women with missing data, 1990 postmenopausal women remained for inclusion in the present analyses (Fig. S2).

The mean (SD) age at the time of DXA imaging was 60.6 (5.8) years (Table 1). Overall, 30% of women reported their ethnicity as Malay, 53% as Chinese, and 17% as Indian. The mean (SD) BMD was 1.08 (0.11) g/cm^2^ for the whole-body total, 0.96 (0.15) g/cm^2^ for the lumbar spine, 2.21 (0.22) g/cm^2^ for the leg, and 1.36 (0.12) g/cm^2^ for the arm. The mean (SD) total body fat percentage was 37.8% (5.8%) and was 7.1% (0.92%) for subcutaneous fat percentage, 2.2% (0.62%) for visceral fat percentage, and 16.9% (2.4%) for gynoid fat percentage.

**Table 1:** Participant characteristics by quintiles of total body fat percentage

Characteristics	Quintile 1	Quintile 2	Quintile 3	Quintile 4	Quintile 5	Total
Total body fat (%)	29.5 (3.1)	34.8 (1.0)	37.9 (0.8)	40.9 (0.9)	45.8 (2.6)	37.8 (5.8)
**Sociodemographic factors**						
Age at DXA scan (years)	60.8 (6.1)	60.7 (5.8)	61.2 (5.7)	60.6 (5.7)	59.6 (5.5)	60.6 (5.8)
Ethnicity						
* Malay*	60 (15.1)	89 (22.4)	140 (35.2)	152 (38.2)	160 (40.2)	601 (30.2)
*Chinese*	327 (82.2)	275 (69.1)	205 (51.5)	157 (39.4)	85 (21.4)	1049 (52.7)
*Indian*	11 (2.8)	34 (8.5)	53 (13.3)	89 (22.4)	153 (38.4)	340 (17.1)
Education						
*Primary*	55 (13.8)	64 (16.1)	68 (17.1)	50 (12.6)	52 (13.1)	289 (14.5)
*Secondary*	208 (52.3)	205 (51.5)	202 (50.8)	215 (54.0)	233 (58.5)	1063 (53.4)
*Tertiary*	135 (33.9)	129 (32.4)	128 (32.2)	133 (33.4)	113 (28.4)	638 (32.1)
**Medical and reproductive history**						
Diabetes^a^	94 (23.6)	102 (25.6)	112 (28.1)	151 (37.9)	140 (35.2)	599 (30.1)
Years since last menses	10.5 (7.2)	10.1 (6.3)	10.6 (6.8)	9.9 (6.1)	9.3 (6.3)	10.1 (6.6)
HRT use	11 (2.8)	10 (2.5)	11 (2.8)	6 (1.5)	15 (3.8)	53 (2.7)
Oral contraceptive use	60 (15.1)	67 (16.8)	88 (22.1)	78 (19.6)	92 (23.1)	385 (19.3)
Number of pregnancies	3 (2)	3 (2)	3 (2)	3 (2)	4 (2)	3 (2)
**Anthropometric measures**						
Height (cm)	156 (6)	156 (5)	155 (6)	155 (5)	154 (6)	155 (5)
BMI (kg/m^2^)	21.1 (2.7)	23.8 (2.8)	25.3 (3.1)	27.3 (3.6)	31.4 (4.7)	25.8 (4.9)
Waist circumference (cm)	72 (8)	79 (8)	83 (9)	86 (9)	93 (10)	83 (11)
**DXA measures**						
Subcutaneous fat (%)^b^	7.5 (0.9)	7.3 (0.9)	7.1 (0.9)	6.9 (0.9)	6.7 (0.8)	7.1 (0.9)
Visceral fat (%)^b^	2.1 (0.7)	2.3 (0.6)	2.3 (0.6)	2.3 (0.6)	2.2 (0.5)	2.2 (0.6)
Gynoid fat (%)^b^	18.6 (2.8)	17.1 (2.5)	16.6 (2.0)	16.0 (1.9)	15.9 (1.7)	16.9 (2.4)
Lean mass (kg)	34.5 (4.4)	35.9 (4.6)	35.7 (5.1)	36.8 (5.6)	38.2 (5.6)	36.2 (5.2)

Results shown are mean (SD) or *N* (%)

*BMI* body mass index, *DXA* dual-energy X-ray absorptiometry, *HRT* hormone replacement therapy

^a^Diabetes defined as previous diagnosis or HbA1c levels > 6.3%

^b^Regional fat presented as a percentage of total body fat

On average, women in the highest fifth of total body fat percentage tended to be younger, more likely to be Indian or Malay than Chinese, had a greater number of pregnancies, and were more likely to have diabetes. Additionally, those with higher total body fat percentages tended to have higher lean mass, BMI, and waist circumference (Table 1). Women within the highest fifth of subcutaneous and gynoid fat percentage were more likely to be Chinese, have a lower BMI, and were less likely to have diabetes (Tables S1 and S2). The same trends were seen in women in the highest fifth of visceral fat percentage except they were more likely to have a higher BMI (Table S3).

### Associations of total and regional body fat percentage with BMD

**Fig. 1:**
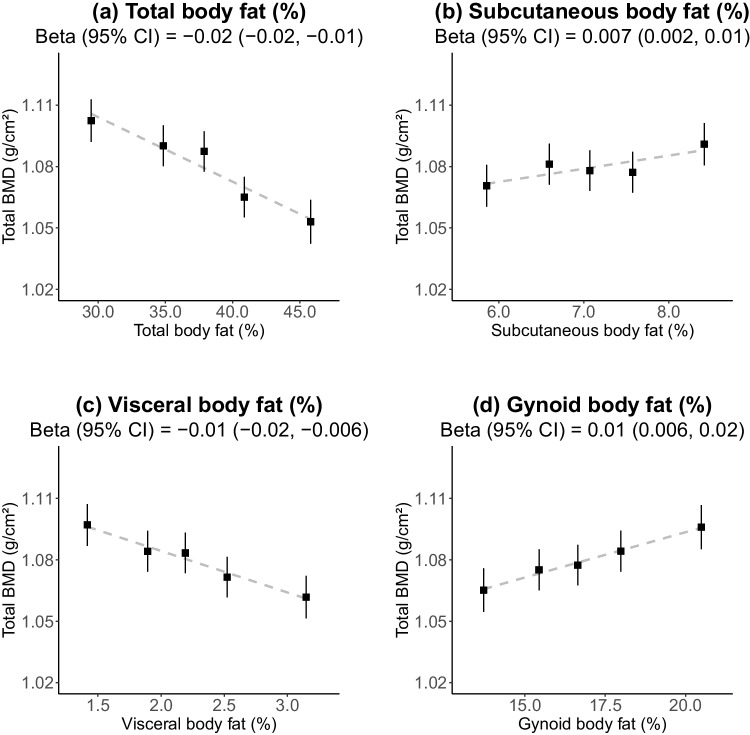
Association of total bone mineral density with **a** total, **b** subcutaneous, **c** visceral, and **d** gynoid body fat percentage. Adjusted for age, height, lean mass, ethnicity, education, and diabetes. Error bars refer to 95% confidence intervals. X-axis scaled to 3.25 standard deviations. Y-axis scaled to 1 standard deviation

There was no evidence of an association between total body fat and total BMD, given age and height. However, given lean mass, the association became inverse. With further adjustment for ethnicity, education, and diabetes, the association of total body fat percentage and total BMD remained inverse (Fig. S3). Each 1 SD higher total and visceral body fat percentage was associated with − 0.02 (95% CI − 0.02, − 0.01) g/cm^2^ and − 0.01 (− 0.02, − 0.006) g/cm^2^ lower total BMD, respectively (Fig. 1). Meanwhile, weak positive associations with total BMD were observed for gynoid (0.01 [0.006, 0.02] g/cm^2^) and subcutaneous (0.007 [0.002, 0.01] g/cm^2^) fat percentages.

**Fig. 2:**
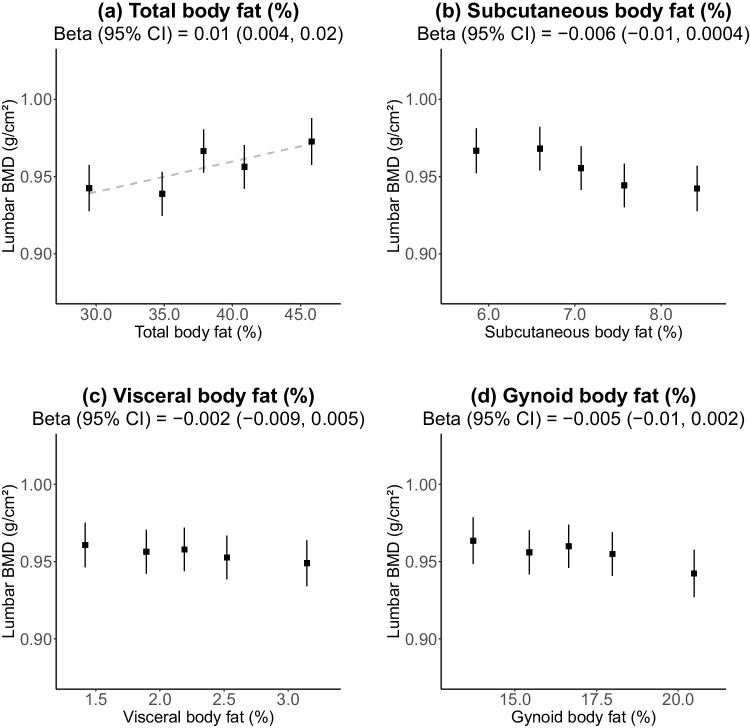
Association of lumbar bone mineral density with **a** total, **b** subcutaneous, **c** visceral, and **d** gynoid body fat percentage. Adjusted for age, height, lean mass, ethnicity, education, and diabetes. Error bars refer to 95% confidence intervals. X-axis scaled to 3.25 standard deviations. Y-axis scaled to 1 standard deviation

There was a modest positive association between total body fat percentage and BMD in the lumbar region, with each 1 SD higher total body fat percentage associated with 0.01 (95% CI 0.004, 0.02) g/cm^2^ higher BMD (Fig. 2). Regional fat percentages (subcutaneous, visceral, and gynoid) were not associated with lumbar BMD.

**Fig. 3:**
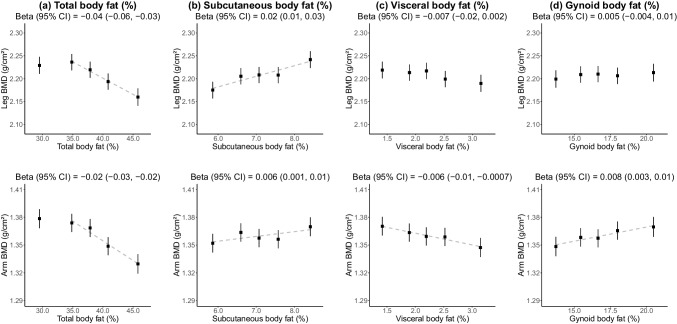
Association of leg and arm BMD with **a** total, **b** subcutaneous, **c** visceral, and **d** gynoid body fat percentage. Adjusted for age, height, lean mass, ethnicity, education, and diabetes. Error bars refer to 95% confidence intervals. X-axis scaled to 3.25 standard deviations. Y-axis scaled to 1 standard deviation

For total body fat percentages greater than the lowest quintile (< 33.0%), each 1 SD higher body fat percentage was inversely associated with BMD of the leg (− 0.04 [95% CI − 0.06, − 0.03] g/cm^2^) and arm (− 0.02 [− 0.03, − 0.02] g/cm^2^) (Fig. 3). A more modest inverse association was observed between visceral body fat percentage and arm BMD; each 1 SD higher was associated with 0.006 (95% CI 0.001, 0.01) g/cm^2^ lower BMD. In contrast, apparent positive associations with arm BMD were observed for subcutaneous fat percentage (0.006 [0.001, 0.01] g/cm^2^) and gynoid fat percentage (0.008 [0.003, 0.01] g/cm^2^). Subcutaneous fat percentage was also positively associated with leg BMD (0.02 [0.01, 0.03] g/cm^2^), but there was no evidence of associations of visceral or gynoid fat percentage with leg BMD.

### Effect modification

There was no evidence of heterogeneity in the association between total body fat percentage and total BMD by ethnicity or diabetes nor trend by years since last menses or BMI category (Fig. 4). After correction for multiple testing, there was no significant effect modification for any of the regional measures of adiposity (FDR = 0.003) (Fig. S4–S7).

**Figure:**
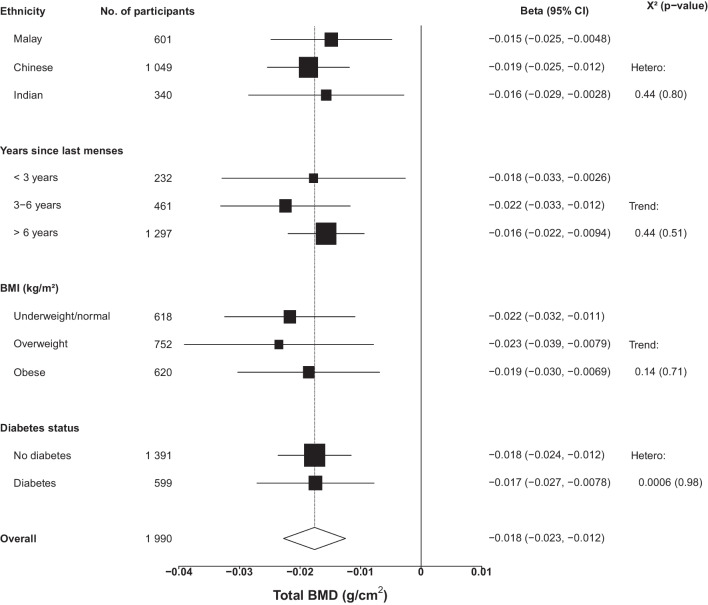
Association of total body fat percentage with total bone mineral density by ethnicity, years since last menses, BMI, and diabetes status**.** Box sizes are weighted by the standard error, and the horizontal line represents the 95% confidence interval

## Discussion

With approximately 2000 women of Malay, Chinese, or Indian ethnicity in Malaysia, this cross-sectional study of the associations of DXA-derived measures of total and regional body fat percentage with total and regional BMD is the largest multi-ethnic comparison among postmenopausal women to date. We found that higher total body fat percentage was independently associated with lower total and regional BMD, with the exception of BMD in the lumbar spine for which there was a positive association. There was an inverse association of visceral body fat percentage and a positive association of gynoid body fat percentage with total BMD, but limited evidence of associations between the other assessed measures of regional fat and BMD. Associations of total and regional body fat percentages with total BMD did not differ across ethnic groups or by menopausal years, BMI category, or diabetes status.

The inverse associations of total body fat percentage with total, arm, and leg BMD are in contrast with findings from many previous studies. For instance, two cross-sectional studies of approximately 400 postmenopausal women in China concluded that total percent fat was positively associated with BMD of the whole-body and individual regions [10, 11]. However, these studies were limited by their low statistical power and a restrictive sample criterion of only normal and overweight (but not obese) women [10]. A study of 1448 postmenopausal Thai women found that after accounting for lean mass, there was a negative, albeit small, association of total fat mass with total BMD, and positive associations with BMD of the lumbar spine and femur [23]. A study of 727 postmenopausal women in Korea similarly reported inverse associations of total body fat percentage with hip and arm BMD but, also unlike the current study, found no significant association with lumbar BMD [2]. This study also found that total body fat mass (rather than percentage) was positively associated with BMD at all three sites, but this was not adjusted for lean mass [2]. In previous literature, lean mass has been found to be positively associated with BMD, and sequential model building in the current analysis demonstrated that positive associations between total body fat and BMD became inverse after adjustment for lean mass [10–12]. Although previous literature has shown positive associations between adiposity and BMD, they have either not adjusted for lean mass or have instead used BMI. Given the established impact of lean mass on the association of adiposity with BMD, these findings may reflect the confounding influence of lean mass and not the actual impact of fat mass. This is particularly relevant in studies using BMI, as it combines lean mass, fat mass, and bone mass into a single measure meant to represent adiposity.

Research on the association of regional fat and BMD in postmenopausal women has been limited. Earlier work focused on the android-to-gynoid ratio as a proxy for the regional distribution of fat in postmenopausal women [10, 11]. Two small cross-sectional studies in China both found that the android-to-gynoid fat mass ratio was inversely associated with total, leg, and arm BMD, but had no association with lumbar spine BMD [10, 11]. This is consistent with the current study’s findings that a higher proportion of body fat in the visceral region, which is inherently a higher proportion of fat in the android region, is inversely associated with total, leg, and arm BMD. It is also aligned with the finding that a higher gynoid fat percentage is associated positively with total and arm BMD. The android-to-gynoid ratio could reflect both of these observed relationships but cannot distinguish whether the association is due to a higher proportion of android (visceral) fat or a lower proportion of gynoid fat. Using a ratio of different fat regions can conceal potential important differences in regional fat’s association with BMD, differences that were observed in the current study. Future investigations should consider subcutaneous, visceral, android, and gynoid fat separately to avoid masking their independent associations.

More generally, previous research that has investigated the associations of regional fat with total and/or regional BMD has used absolute fat mass (kg) in the region and often has not accounted for the total body fat mass. Consequently, as total body fat mass increases, so does the amount of fat stored in these regions; therefore, associations of regional fat and BMD become biased by overall fat mass. Although some studies have attempted to account for this by adjusting for total body weight [8, 13] or BMI [5], associations with regional fat as a percent of total fat, as in this study, allow for clearer insights into the effects of fat distribution. This shifts the focus from general obesity to the more specific question of how an individual’s fat distribution is associated with their BMD.

This study was unique in being able to study the relevance of ethnicity for the association of overall and regional adiposity for BMD, particularly given the inclusion of an ethnically diverse cohort residing in one country. Ethnicity is an important factor to consider when studying disease associations of adiposity. However, existing literature has been unable to investigate its relevance for the association of adiposity with BMD, since studies have been limited to homogenous samples of predominantly Chinese [10, 11], Korean [2, 13, 15, 17], and European [5, 7, 8, 22, 24] populations. In the current study, no substantial effect modification by ethnicity was observed between total body fat percentage and total BMD. This could reflect a true absence of differences in these associations, but it could also reflect limited statistical power to detect ethnic differences. Further strengths of the study include the sample size; this is the largest study to date to examine the independent relationship between body composition measured by DXA and BMD among postmenopausal women. Additionally, since whole-body total and regional fat measures were examined, the study provides a holistic understanding of the relevance of adiposity for BMD, which appears to differ by the region in which fat is stored.

However, the study has several limitations. Although prior diseases which may impact body composition were excluded, due to the cross-sectional nature of this analysis, reverse causality cannot be ruled out. It is also unclear how generalizable the findings are to other populations as the study was restricted to postmenopausal women from Kuala Lumpur and Selangor. There were also some potential confounders that could not be accounted for, such as steroid use, physical activity, and calcium and vitamin D intake. Also, despite the statistically significant findings, the changes in BMD associated with higher levels of body fat were generally modest compared to the least significant change (the smallest difference in a BMD measure that may be considered a real change) for the DXA systems used [25]. Finally, due to the observational nature of this study, the causality of the observed associations cannot be determined.

## Conclusion

Among postmenopausal women in Malaysia total body fat percentage was inversely associated with total, leg, and arm BMD, but positively associated with lumbar BMD, while associations between regional fat percentages and BMD differed depending on the locality. An understanding of the relationships of total and regional adiposity with BMD could help identify at-risk individuals who are currently overlooked as the only body composition measure used by clinicians to assess osteoporosis and fracture risk is BMI [26]. Although reported findings are small, with the spread of the obesity epidemic, these higher values of adiposity that are associated with lower BMD are becoming more common and could potentially have larger impacts at the population level. The findings from the current study need to be confirmed in large, ethnically diverse samples, utilizing prospective data to examine adiposity’s impact on incident clinical outcomes such as osteopenia, osteoporosis, and fractures. By understanding how adiposity impacts bone formation and loss, health systems can act earlier to prevent the rising global burden of disability and fractures attributable to low BMD.

## Supplementary Information

Below is the link to the electronic supplementary material.

Supplementary file1 (PDF 365 KB)

## Data Availability

The datasets used and/or analyzed during the current study are available from the corresponding author on reasonable request.

## Footnotes

1 Regional fat as a percentage of total fat is used in both statistical analysis and presented in figures for subcutaneous, visceral, and gynoid fat measures.
