# Supplementary material for: Body fat distribution and bone mineral density in a multi-ethnic sample of postmenopausal women in The Malaysian Cohort
Source: Arch Osteoporos. 2024 Aug 7;19(1):73. doi: 10.1007/s11657-024-01435-x (PMC11306509; doi:10.1007/s11657-024-01435-x)
Supplement: Supplementary file 1 — Supplementary file1 (PDF 365 KB) [file 11657_2024_1435_MOESM1_ESM.pdf]

Online Resource 1: Supplementary Appendix

Table of Contents

**Table SI1**	Participant characteristics by quintiles of subcutaneous fat as a percentage of total body fat	Page 2
**Table SI2**	Participant characteristics by quintiles of gynoid fat as a percentage of total body fat	Page 3
**Table SI3**	Participant characteristics by quintiles of visceral fat as a percentage of total body fat	Page 4
**Figure SI1**	Whole body DXA scan regional division	Page 5
**Figure SI2**	Participant exclusions to derive sample population	Page 6
**Figure SI3**	Association of total body fat percentage with total bone mineral density with sequential adjustment for potential confounders	Page 7
**Figure SI4**	Association of subcutaneous body fat percentage with total bone mineral density by ethnicity, years since last menses, BMI and diabetes status	Page 8
**Figure SI5**	Association of gynoid body fat percentage with total bone mineral density by ethnicity, years since last menses, BMI and diabetes status	Page 9
**Figure SI6**	Association of visceral body fat percentage with total bone mineral density by ethnicity, years since last menses, BMI and diabetes status	Page 10
**Figure SI7**	Association of total BMD with (a) total, (b) subcutaneous, (c) visceral and (d) gynoid body fat percentage by ethnicity	Page 11

Table SI1. Participant characteristics by quintiles of subcutaneous fat as a percentage of total body fat

Characteristics	Quintile 1	Quintile 2	Quintile 3	Quintile 4	Quintile 5	Total
Subcutaneous fat (%)[a]	5.9 (0.4)	6.6 (0.2)	7.1 (0.1)	7.6 (0.2)	8.4 (0.5)	7.1 (0.9)
**SOCIODEMOGRAPHIC FACTORS**						
Age at DXA scan (years)	59.6 (5.6)	59.8 (5.7)	60.3 (5.6)	60.9 (5.8)	62.2 (5.9)	60.6 (5.8)
Ethnicity						
Malay	136 (34.2)	122 (30.7)	121 (30.4)	111 (27.9)	111 (27.9)	601 (30.2)
Chinese	177 (44.5)	183 (46.0)	226 (56.8)	236 (59.3)	227 (57.0)	1,049 (52.7)
Indian	85 (21.4)	93 (23.4)	51 (12.8)	51 (12.8)	60 (15.1)	340 (17.1)
Education						
Primary	54 (13.6)	51 (12.8)	60 (15.1)	58 (14.6)	66 (16.6)	289 (14.5)
Secondary	207 (52.0)	207 (52.0)	217 (54.5)	215 (54.0)	217 (54.5)	1,063 (53.4)
Tertiary	137 (34.4)	140 (35.2)	121 (30.4)	125 (31.4)	115 (28.9)	638 (32.1)
**MEDICAL AND REPRODUCTIVE HISTORY**						
Years since stopped menses	9.0 (6.4)	9.8 (6.9)	9.9 (6.0)	10.4 (6.7)	11.3 (6.6)	10.1 (6.6)
HRT use	9 (2.3)	16 (4.0)	7 (1.8)	11 (2.8)	10 (2.5)	53 (2.7)
Oral contraceptive use	79 (19.8)	80 (20.1)	69 (17.3)	78 (19.6)	79 (19.8)	385 (19.3)
Number of pregnancies	3 (2)	3 (2)	3 (2)	3 (2)	3 (2)	3 (2)
Diabetes[b]	140 (35.2)	121 (30.4)	107 (26.9)	110 (27.6)	121 (30.4)	599 (30.1)
**ANTHROMETRIC MEASURES**						
Height (cm)	157 (5)	156 (5)	156 (5)	154 (5)	152 (5)	155 (5)
BMI (kg/m^2)	27.8 (5.6)	26.7 (5.1)	25.6 (4.4)	25.0 (4.2)	23.9 (4.0)	25.8 (4.9)
Waist circumference (cm)	85 (12)	84 (12)	83 (11)	82 (10)	80 (10)	83 (11)
**DXA MEASURES**						
Total body fat (%)	39.9 (6.0)	39.2 (5.8)	37.6 (5.4)	36.8 (5.3)	35.4 (5.6)	37.8 (5.8)
Visceral fat (%)[a]	2.1 (0.6)	2.2 (0.6)	2.3 (0.6)	2.3 (0.6)	2.4 (0.7)	2.2 (0.6)
Gynoid fat (%)[a]	17.2 (2.4)	16.9 (2.4)	16.9 (2.5)	16.8 (2.3)	16.5 (2.6)	16.9 (2.4)
Lean mass (kg)	38.6 (6.0)	36.9 (5.1)	36.4 (4.8)	35.5 (4.4)	33.7 (4.3)	36.2 (5.2)

BMI, body mass index; DXA, dual-energy X-ray absorptiometry; HRT, hormone replacement therapy
Results shown are mean (SD) or N (%)
[a]Regional fat presented as a percentage of total body fat
[b]Diabetes defined as previous diagnosis or HbA1c levels > 6.3%

Table SI2. Participant characteristics by quintiles of gynoid fat as a percentage of total body fat

Characteristics	Quintile 1	Quintile 2	Quintile 3	Quintile 4	Quintile 5	Total
Gynoid fat (%)[a]	13.7 (0.9)	15.4 (0.3)	16.6 (0.4)	18.0 (0.4)	20.5 (1.4)	16.9 (2.4)
**SOCIODEMOGRAPHIC FACTORS**						
Age at DXA scan (years)	61.6 (5.5)	61.1 (5.6)	60.5 (5.9)	60.4 (5.7)	59.2 (5.9)	60.6 (5.8)
Ethnicity						
Malay	180 (45.2)	151 (37.9)	114 (28.6)	100 (25.1)	56 (14.1)	601 (30.2)
Chinese	126 (31.7)	167 (42.0)	207 (52.0)	238 (59.8)	311 (78.1)	1,049 (52.7)
Indian	92 (23.1)	80 (20.1)	77 (19.3)	60 (15.1)	31 (7.8)	340 (17.1)
Education						
Primary	68 (17.1)	60 (15.1)	64 (16.1)	52 (13.1)	45 (11.3)	289 (14.5)
Secondary	221 (55.5)	222 (55.8)	204 (51.3)	211 (53.0)	205 (51.5)	1,063 (53.4)
Tertiary	109 (27.4)	116 (29.1)	130 (32.7)	135 (33.9)	148 (37.2)	638 (32.1)
**MEDICAL AND REPRODUCTIVE HISTORY**						
Years since stopped menses	10.9 (6.6)	10.6 (6.3)	9.9 (6.7)	10.0 (6.4)	8.9 (6.6)	10.1 (6.6)
HRT use	12 (3.0)	9 (2.3)	7 (1.8)	9 (2.3)	16 (4.0)	53 (2.7)
Oral contraceptive use	107 (26.9)	81 (20.4)	75 (18.8)	69 (17.3)	53 (13.3)	385 (19.3)
Number of pregnancies	4 (2)	4 (2)	3 (2)	3 (2)	3 (2)	3 (2)
Diabetes[b]	222 (55.8)	152 (38.2)	110 (27.6)	83 (20.9)	32 (8.0)	599 (30.1)
**ANTHROMETRIC MEASURES**						
Height (cm)	154 (5)	154 (6)	155 (5)	156 (5)	157 (5)	155 (5)
BMI (kg/m^2)	28.3 (4.8)	27.3 (4.6)	26.4 (4.7)	25.0 (4.1)	22.0 (3.5)	25.8 (4.9)
Waist circumference (cm)	91 (9)	86 (10)	83 (10)	80 (9)	72 (8)	83 (11)
**DXA MEASURES**						
Total body fat (%)	39.6 (4.9)	39.6 (4.9)	38.8 (5.6)	37.7 (5.2)	33.2 (6.0)	37.8 (5.8)
Subcutaneous fat (%)[a]	7.3 (0.9)	7.2 (0.9)	7.0 (0.9)	7.0 (0.9)	7.1 (0.9)	7.1 (0.9)
Visceral fat (%)[a]	2.7 (0.6)	2.4 (0.6)	2.3 (0.5)	2.1 (0.5)	1.8 (0.5)	2.2 (0.6)
Lean mass (kg)	38.1 (5.2)	36.9 (5.5)	36.5 (5.4)	35.7 (4.8)	33.9 (4.3)	36.2 (5.2)

BMI, body mass index; DXA, dual-energy X-ray absorptiometry; HRT, hormone replacement therapy
Results shown are mean (SD) or N (%)
[a]Regional fat presented as a percentage of total body fat
[b]Diabetes defined as previous diagnosis or HbA1c levels > 6.3%

Table SI3. Participant characteristics by quintiles of visceral fat as a percentage of total body fat

Characteristics	Quintile 1	Quintile 2	Quintile 3	Quintile 4	Quintile 5	Total
Visceral fat (%)[a]	1.4 (0.3)	1.9 (0.1)	2.2 (0.1)	2.5 (0.1)	3.1 (0.4)	2.2 (0.6)
**SOCIODEMOGRAPHIC FACTORS**						
Age at DXA scan (years)	59.8 (6.1)	59.8 (5.8)	59.9 (5.4)	61.0 (5.4)	62.4 (5.8)	60.6 (5.8)
Ethnicity						
Malay	126 (31.7)	139 (34.9)	119 (29.9)	116 (29.1)	101 (25.4)	601 (30.2)
Chinese	204 (51.3)	176 (44.2)	207 (52.0)	213 (53.5)	249 (62.6)	1,049 (52.7)
Indian	68 (17.1)	83 (20.9)	72 (18.1)	69 (17.3)	48 (12.1)	340 (17.1)
Education						
Primary	43 (10.8)	53 (13.3)	54 (13.6)	54 (13.6)	85 (21.4)	289 (14.5)
Secondary	211 (53.0)	203 (51.0)	215 (54.0)	217 (54.5)	217 (54.5)	1,063 (53.4)
Tertiary	144 (36.2)	142 (35.7)	129 (32.4)	127 (31.9)	96 (24.1)	638 (32.1)
**MEDICAL AND REPRODUCTIVE HISTORY**						
Years since stopped menses	9.6 (6.9)	9.3 (6.6)	9.3 (6.1)	10.5 (6.4)	11.6 (6.5)	10.1 (6.6)
HRT use	15 (3.8)	8 (2.0)	17 (4.3)	8 (2.0)	5 (1.3)	53 (2.7)
Oral contraceptive use	65 (16.3)	76 (19.1)	82 (20.6)	76 (19.1)	86 (21.6)	385 (19.3)
Number of pregnancies	3 (2)	3 (2)	3 (2)	3 (2)	3 (2)	3 (2)
Diabetes[b]	57 (14.3)	94 (23.6)	103 (25.9)	147 (36.9)	198 (49.7)	599 (30.1)
**ANTHROMETRIC MEASURES**						
Height (cm)	156 (6)	155 (6)	156 (6)	155 (5)	154 (5)	155 (5)
BMI (kg/m^2)	24.1 (5.6)	25.8 (5.0)	25.8 (4.8)	26.5 (4.3)	26.7 (4.2)	25.8 (4.9)
Waist circumference (cm)	77 (12)	82 (11)	83 (11)	85 (10)	87 (9)	83 (11)
**DXA MEASURES**						
Total body fat (%)	36.4 (7.4)	38.2 (6.0)	38.5 (5.4)	38.4 (4.9)	37.4 (4.9)	37.8 (5.8)
Subcutaneous fat (%)[a]	6.9 (1.0)	7.1 (0.9)	7.1 (0.9)	7.1 (0.9)	7.3 (1.0)	7.1 (0.9)
Gynoid fat (%)[a]	18.6 (2.5)	17.6 (2.2)	16.8 (2.1)	16.2 (1.9)	15.1 (1.8)	16.9 (2.4)
Lean mass (kg)	34.5 (5.4)	36.0 (4.9)	35.9 (4.9)	37.0 (5.1)	37.8 (5.3)	36.2 (5.2)

BMI, body mass index; DXA, dual-energy X-ray absorptiometry; HRT, hormone replacement therapy
Results shown are mean (SD) or N (%)
[a]Regional fat presented as a percentage of total body fat
[b]Diabetes defined as previous diagnosis or HbA1c levels > 6.3%

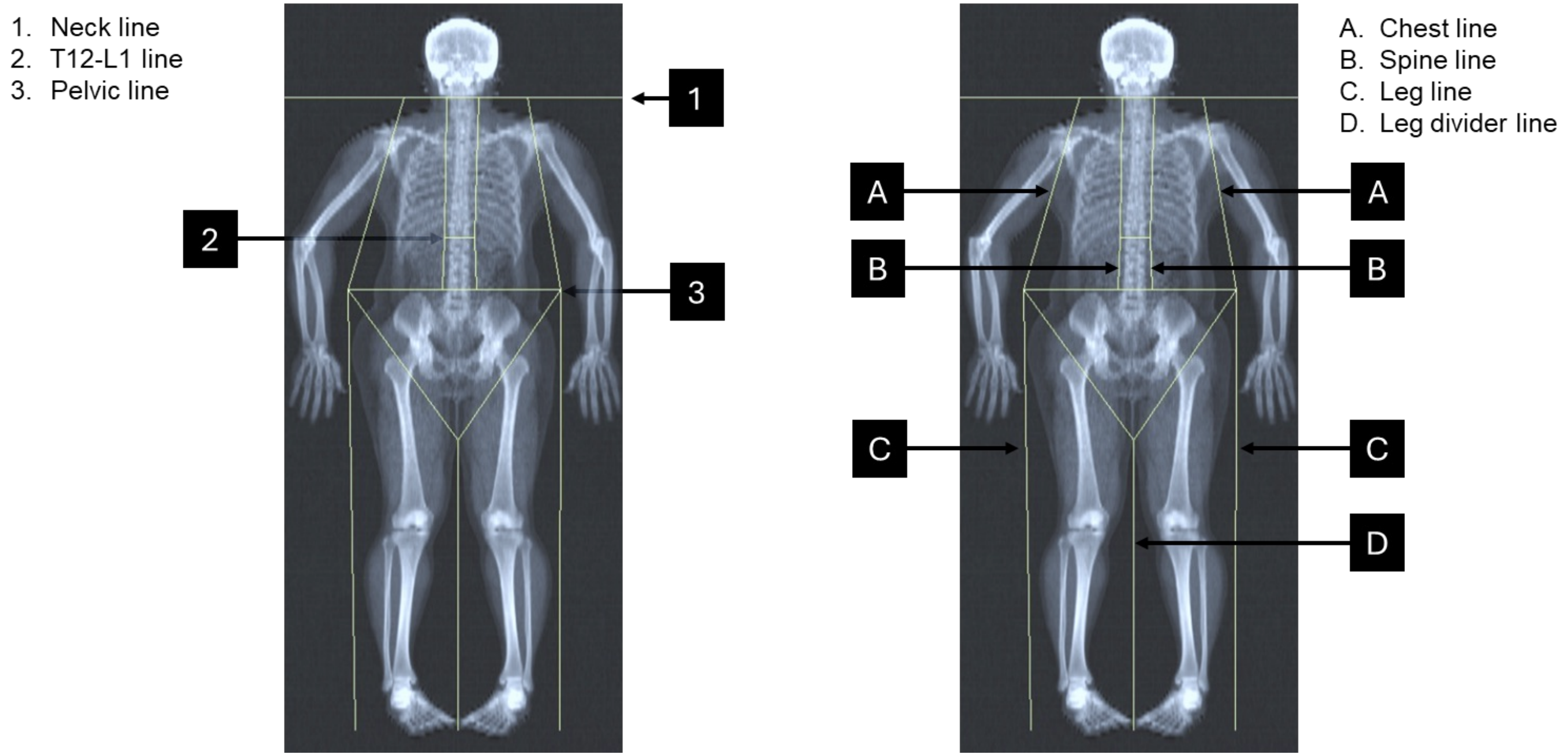

Fig. SI. Whole body DXA scan regional division lines

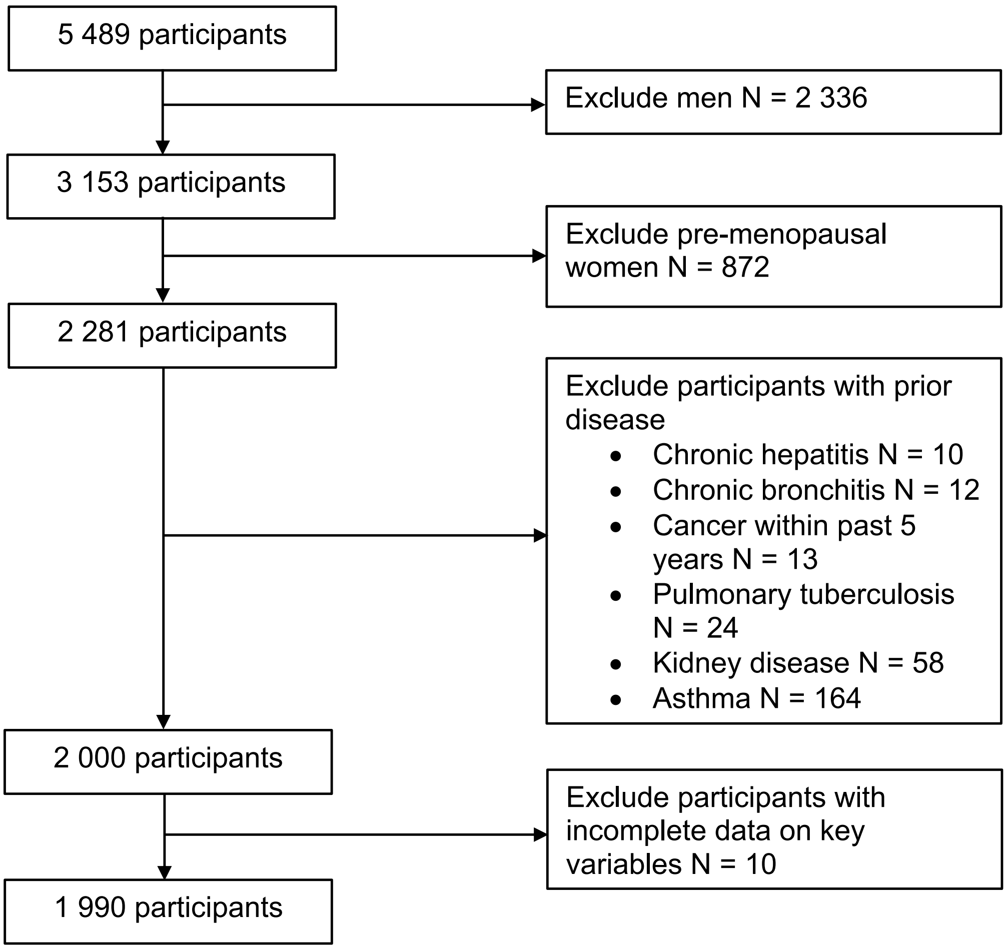

Fig. SI2. Participant exclusions to derive sample population

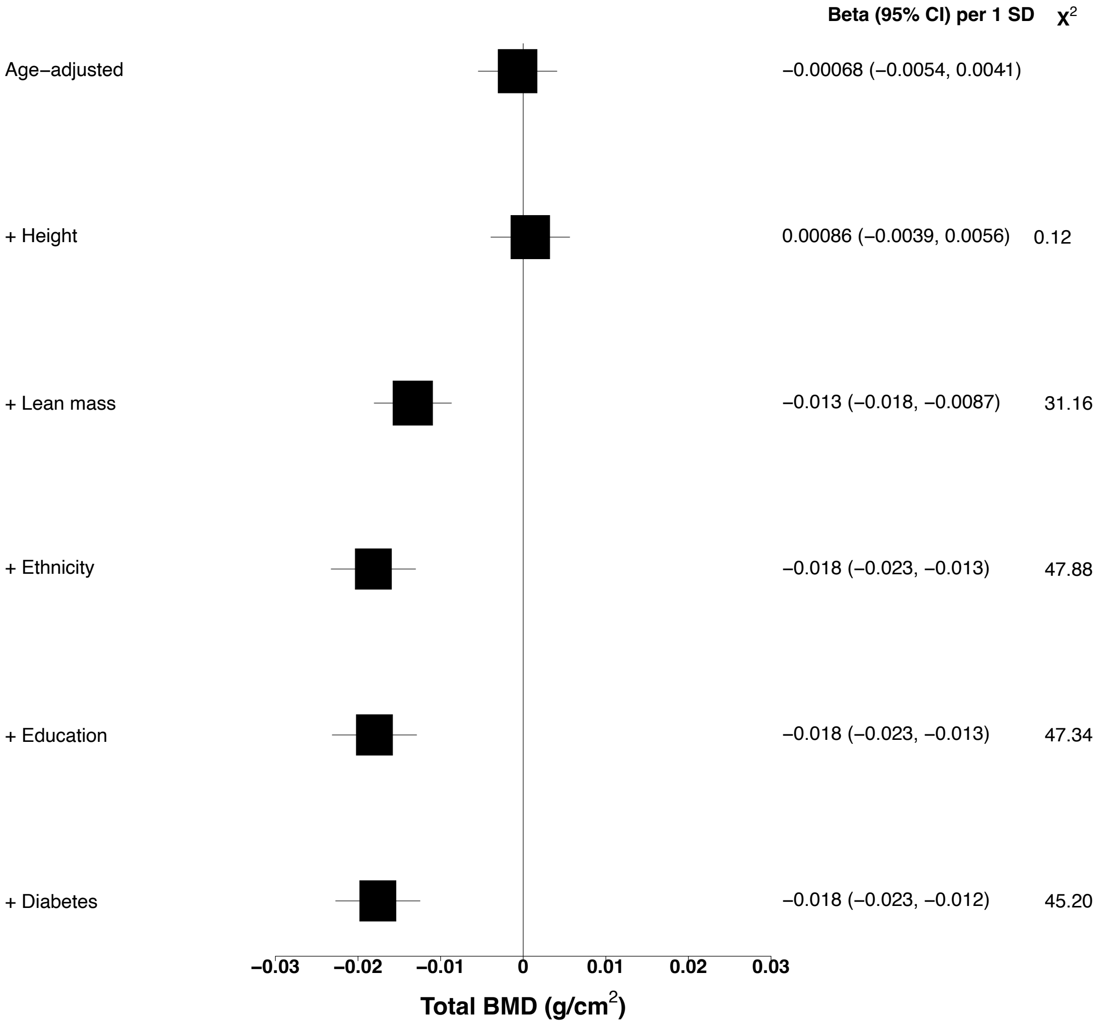

Fig. SI3. Association of total body fat percentage with total bone mineral density with sequential adjustment for potential confounders. Box sizes are weighted by the standard errors and horizontal lines represent the 95% confidence interval

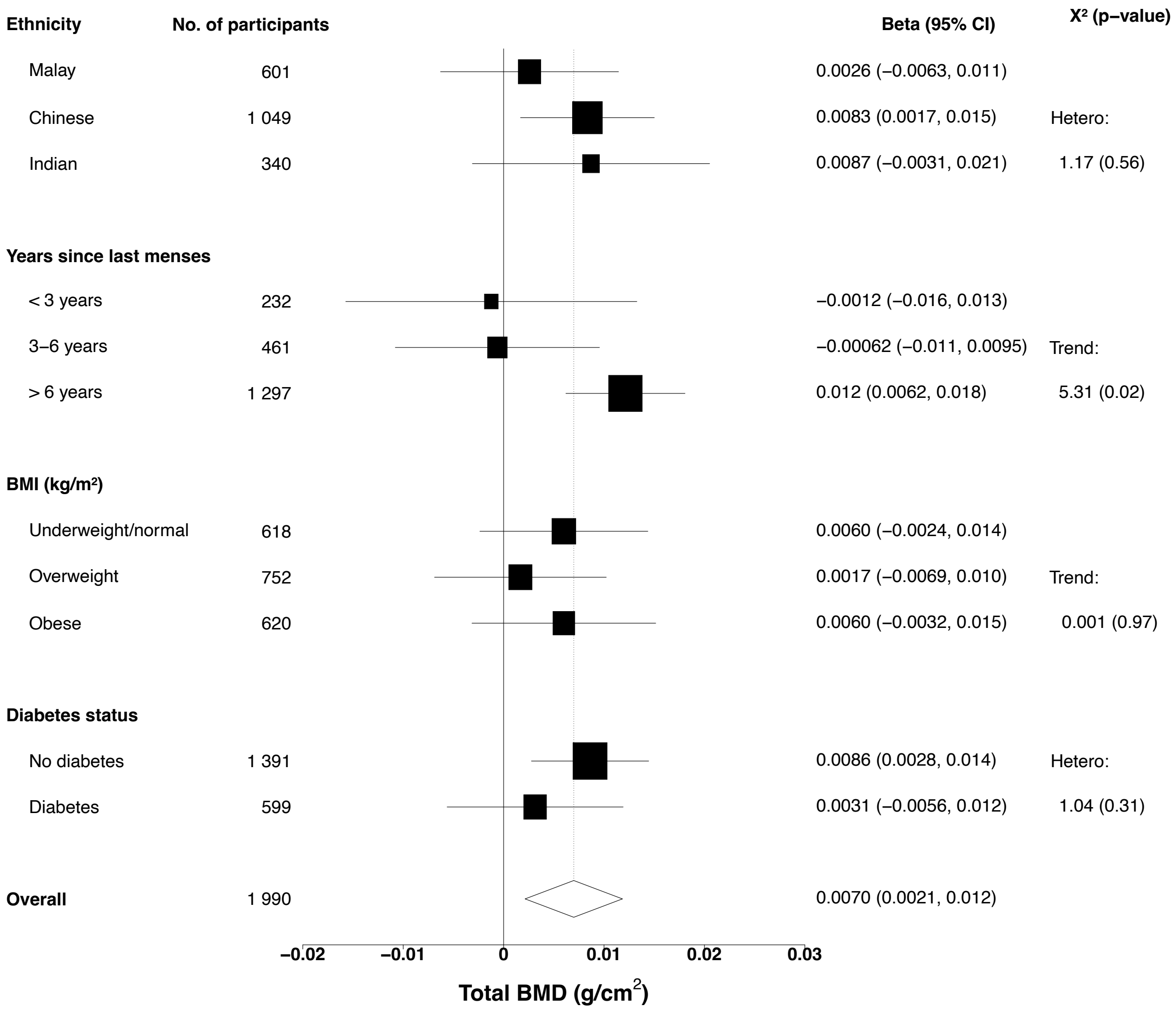

Fig. SI4. Association of subcutaneous body fat percentage with total bone mineral density by ethnicity, years since last menses, BMI and diabetes status. Box sizes are weighted by the standard errors and horizontal lines represent the 95% confidence interval

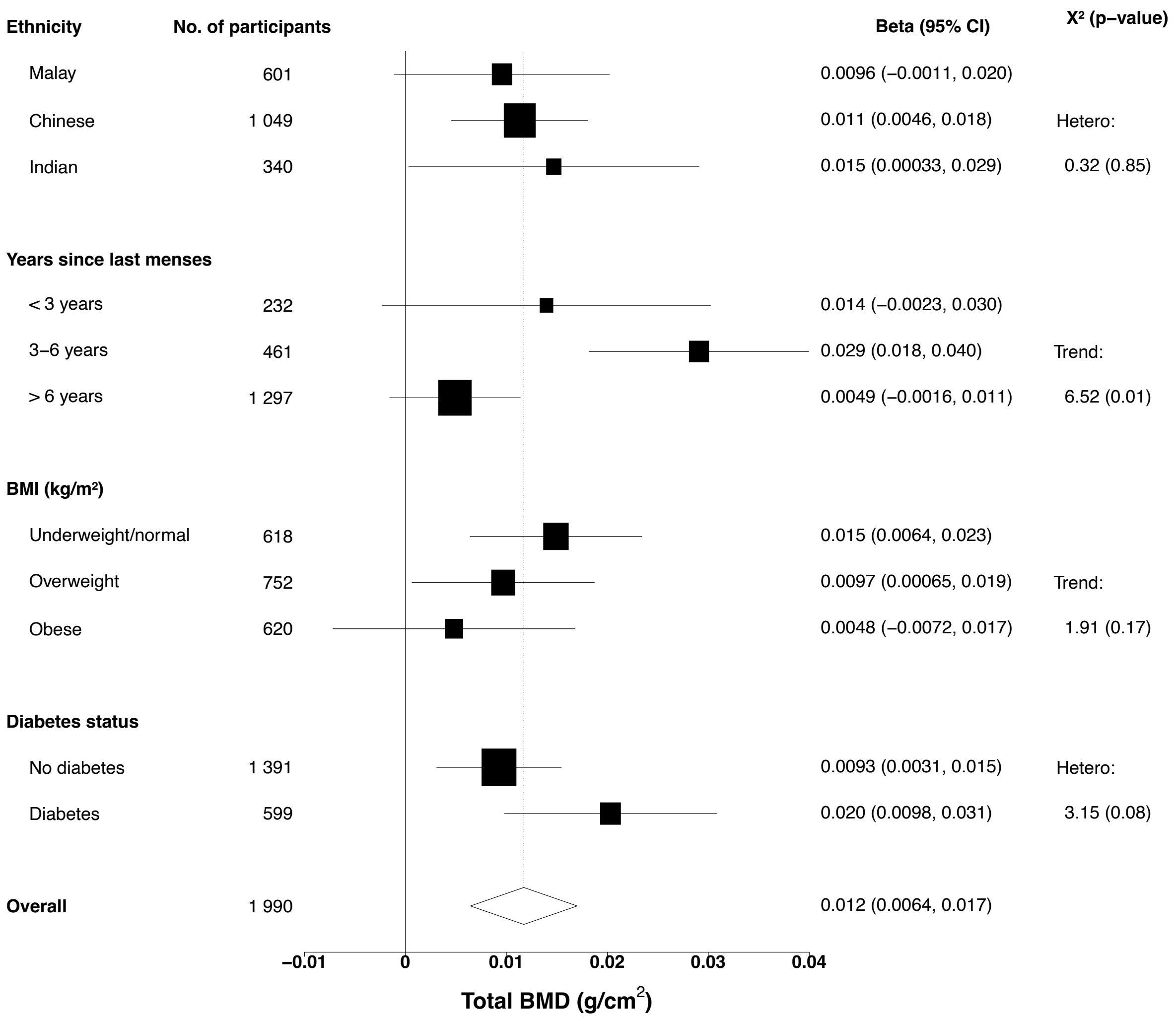

Fig. SI5. Association of gynoid body fat percentage with total bone mineral density by ethnicity, years since last menses, BMI and diabetes status. Box sizes are weighted by the standard errors and horizontal lines represent the 95% confidence intervals

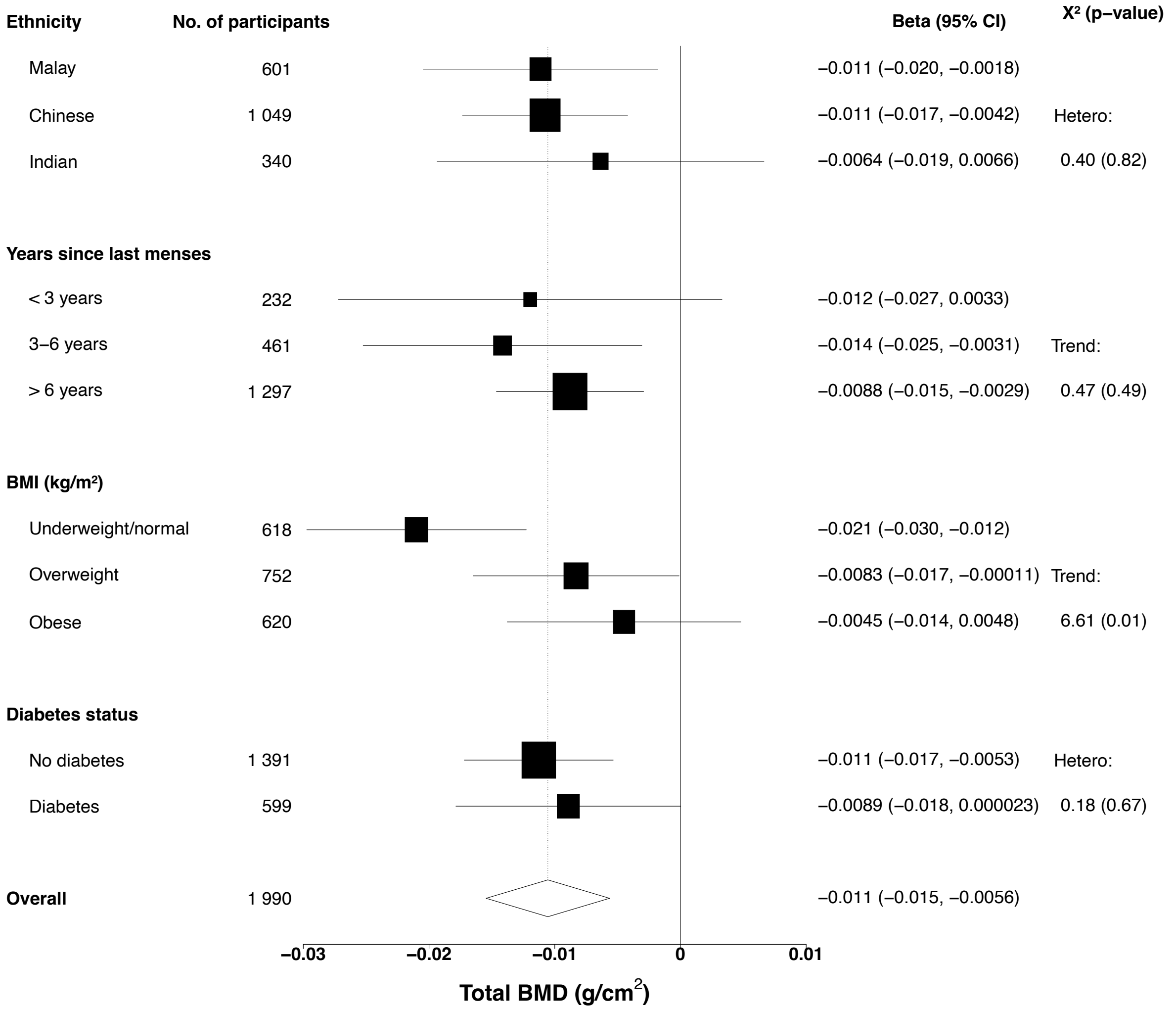

Fig. SI6. Association of visceral body fat percentage with total bone mineral density by ethnicity, years since last menses, BMI and diabetes status. Box sizes are weighted by the standard errors and horizontal lines represent the 95% confidence intervals

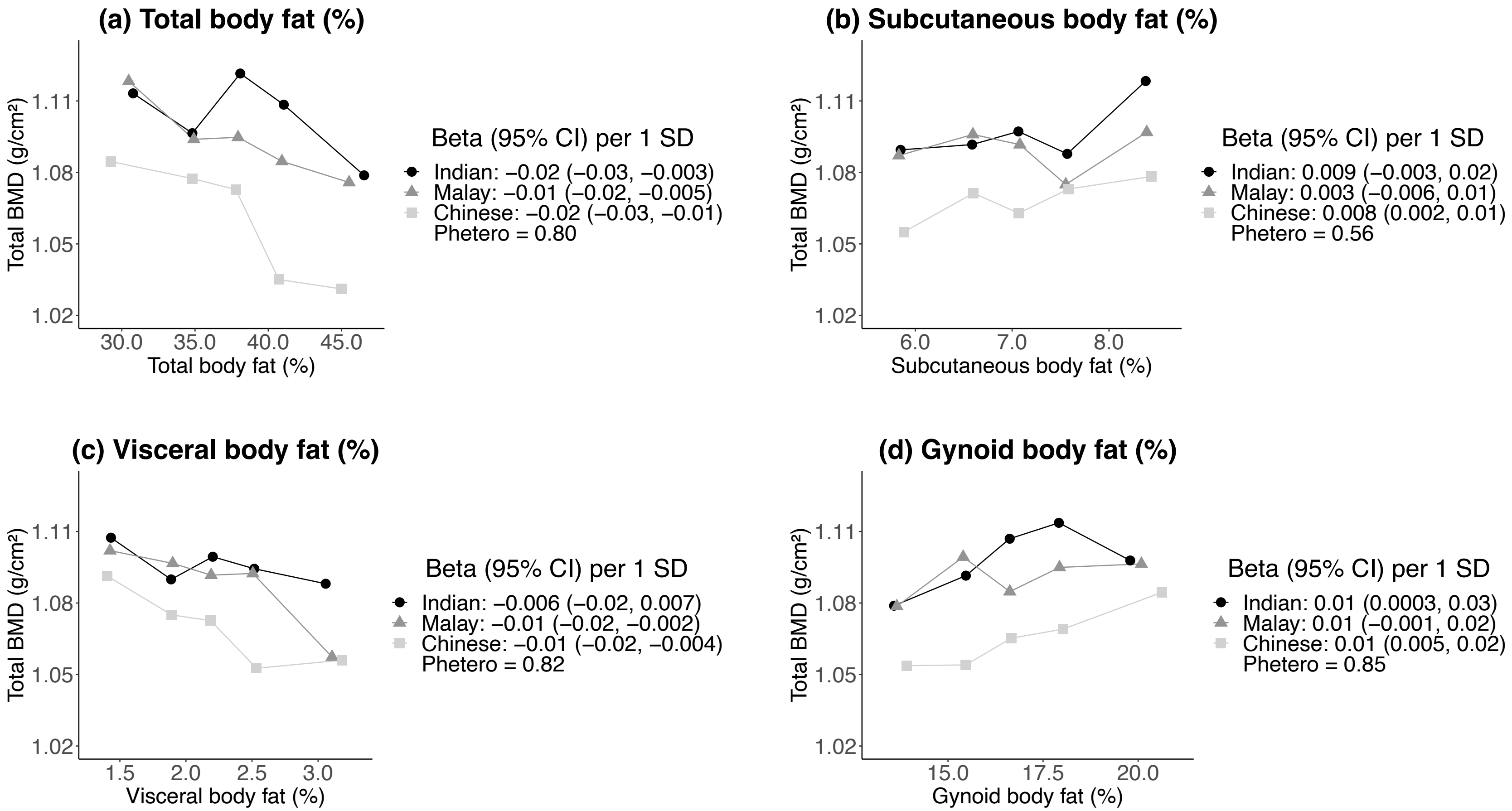

Fig. SI7. Association of total BMD with (a) total, (b) subcutaneous, (c) visceral and (d) gynoid body fat percentage by ethnicity. Adjusted for age, height, lean mass, education and diabetes. Error bars refer to 95% confidence intervals. X-axis scaled to 3.25 standard deviations. Y-axis scaled to 1 standard deviation
